# Ethyl 3-nitro-4-(*n*-propyl­amino)benzoate

**DOI:** 10.1107/S160053680901873X

**Published:** 2009-05-23

**Authors:** Guo-Hua Zhang, Yong-Zhong Wu, Hao-Yuan Li, Bo-Nian Liu, Cheng Guo

**Affiliations:** a College of Science, Nanjing University of Technology, Xinmofan Road No. 5 Nanjing, Nanjing 210009, People’s Republic of China; b Department of Applied Chemistry, Nanjing College of Chemical Technology, Geguan Road No. 625 Dachang District Nanjing, Nanjing 210048, People’s Republic of China; c College of Biotechnology and Pharmaceutical Engineering, Nanjing University of Technology, Xinmofan Road No. 5 Nanjing, Nanjing 210009, People’s Republic of China

## Abstract

In the mol­ecule of the title compound, C_12_H_16_N_2_O_4_, an intra­molecular N—H⋯O hydrogen bond results in the formation of a six-membered ring having an envelope conformation. In the crystal structure, a bifurcated intra/intermolecular N—H⋯(O,O) hydrogen bond generates inversion dimers.

## Related literature

For bond-length data, see: Allen *et al.* (1987 ▶). For the synthesis, see: Ates-Alagoz & Buyukbingol (2001 ▶); Oezden *et al.* (2005 ▶).
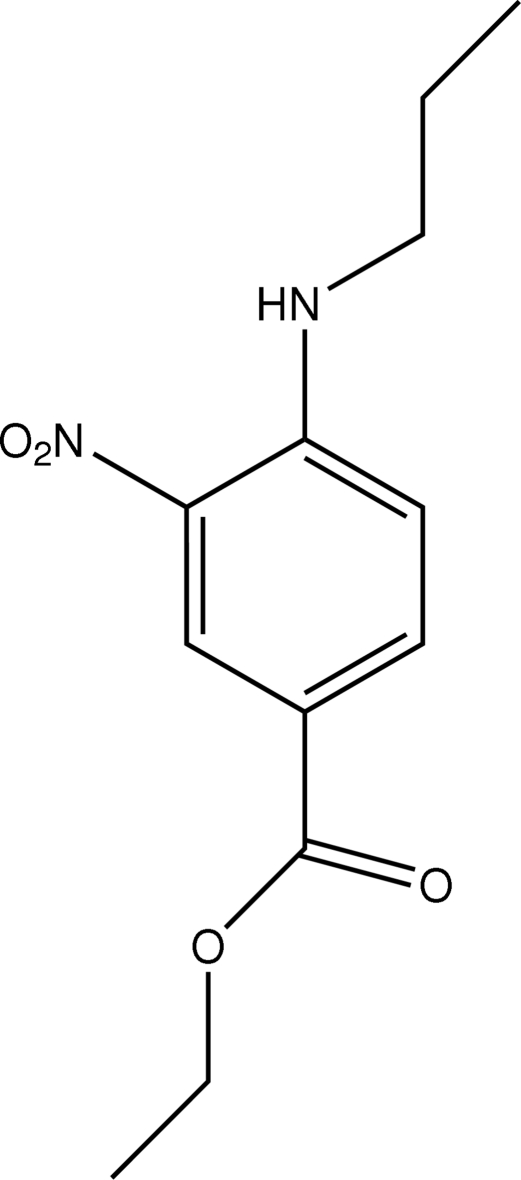


## Experimental

### 

#### Crystal data

C_12_H_16_N_2_O_4_
*M* *_r_* = 252.27
Triclinic, 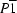

*a* = 4.4400 (9) Å
*b* = 12.606 (3) Å
*c* = 13.209 (3) Å
α = 61.710 (19)°
β = 83.02 (3)°
γ = 81.75 (3)°
*V* = 643.1 (3) Å^3^
*Z* = 2
Mo *K*α radiation
μ = 0.10 mm^−1^
*T* = 294 K
0.20 × 0.10 × 0.10 mm

#### Data collection

Enraf–Nonius CAD-4 diffractometer
Absorption correction: ψ scan (North *et al.*, 1968 ▶) *T*_min_ = 0.981, *T*_max_ = 0.990
2593 measured reflections
2281 independent reflections
924 reflections with *I* > 2σ(*I*)
*R*_int_ = 0.083
3 standard reflections frequency: 120 min intensity decay: 1%

#### Refinement

*R*[*F* ^2^ > 2σ(*F* ^2^)] = 0.067
*wR*(*F* ^2^) = 0.165
*S* = 1.00
2281 reflections
157 parameters
H-atom parameters constrained
Δρ_max_ = 0.19 e Å^−3^
Δρ_min_ = −0.14 e Å^−3^

### 

Data collection: *CAD-4 Software* (Enraf–Nonius, 1989 ▶); cell refinement: *CAD-4 Software*; data reduction: *XCAD4* (Harms & Wocadlo, 1995 ▶); program(s) used to solve structure: *SHELXS97* (Sheldrick, 2008 ▶); program(s) used to refine structure: *SHELXL97* (Sheldrick, 2008 ▶); molecular graphics: *ORTEP-3 for Windows* (Farrugia, 1997 ▶) and *PLATON* (Spek, 2009 ▶); software used to prepare material for publication: *SHELXL97*.

## Supplementary Material

Crystal structure: contains datablocks global, I. DOI: 10.1107/S160053680901873X/hk2690sup1.cif

Structure factors: contains datablocks I. DOI: 10.1107/S160053680901873X/hk2690Isup2.hkl

Additional supplementary materials:  crystallographic information; 3D view; checkCIF report

## Figures and Tables

**Table 1:** Hydrogen-bond geometry (Å, °)

*D*—H⋯*A*	*D*—H	H⋯*A*	*D*⋯*A*	*D*—H⋯*A*
N2—H2*A*⋯O4	0.86	2.02	2.635 (5)	128
N2—H2*A*⋯O4^i^	0.86	2.55	3.324 (6)	150

Symmetry code: (i) 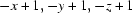.

**Fig. 1.**
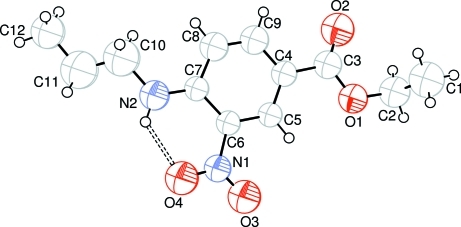
The molecular structure of the title molecule, with the atom-numbering scheme. Hydrogen bond is shown as dashed line.

**Fig. 2.**
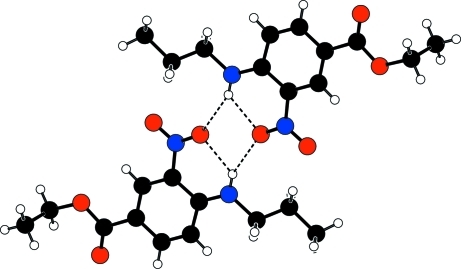
A partial packing diagram of the title compound. Hydrogen bonds are shown as dashed lines.

## supplementary crystallographic information

### Comment

Some derivatives of benzoic acid are important chemical materials. We report herein the crystal structure of the title compound.

In the molecule of the title compound (Fig 1), the bond lengths (Allen *et al.*,  1987) and angles are within normal ranges. Ring A (C4-C9) is, of course, planar. Intramolecular N-H···O hydrogen bond (Table 1) results in the formation of a six-membered ring B (O4/N1/N2/C6/C7/H2A) having envelope conformation with atom O4 displaced by -0.116 (3) Å from the plane of the other ring atoms.

In the crystal structure, intra- and intermolecular N-H···O interactions (Table 1) link the molecules into centrosymmetric dimers (Fig. 2), in which they may be effective in the stabilization of the structure.

### Experimental

For the preparation of the title compound, ethyl 4-chloro-3-nitrobenzoate (5.3 g, 23 mmol) was refluxed in n-propyl amine (25 ml) and tetrahydrofuran (50 ml) for 2 h. Then, solvents were evaporated and water was added to give yellow precipate, which was collected by filtration and washed with cold ethanol (2 × 15 ml) to afford the yellow solid (yield; 4.8 g). Crystals suitable for X-ray analysis were obtained by slow evaporation of an ethanol solution.

### Refinement

H atoms were positioned geometrically, with N-H = 0.86 Å (for NH) and C-H = 0.93, 0.97 and 0.96 Å for aromatic, methylene and methyl H, respectively, and constrained to ride on their parent atoms, with U_iso_(H) = xU_eq_(C,N), where x = 1.5 for methyl H and x = 1.2 for all other H atoms.

### Crystal data

**Table:**

C_12_H_16_N_2_O_4_	*Z* = 2
*M**_r_* = 252.27	*F*(000) = 268
Triclinic, *P*1	*D*_x_ = 1.303 Mg m^−^^3^
Hall symbol: -P 1	Mo *K*α radiation, λ = 0.71073 Å
*a* = 4.4400 (9) Å	Cell parameters from 25 reflections
*b* = 12.606 (3) Å	θ = 9–11°
*c* = 13.209 (3) Å	µ = 0.10 mm^−^^1^
α = 61.710 (19)°	*T* = 294 K
β = 83.02 (3)°	Block, colorless
γ = 81.75 (3)°	0.20 × 0.10 × 0.10 mm
*V* = 643.1 (3) Å^3^	

### Data collection

**Table:**

Enraf–Nonius CAD-4 diffractometer	924 reflections with *I* > 2σ(*I*)
Radiation source: fine-focus sealed tube	*R*_int_ = 0.083
graphite	θ_max_ = 25.2°, θ_min_ = 1.8°
ω/2θ scans	*h* = 0→5
Absorption correction: ψ scan (North *et al.*, 1968)	*k* = −14→15
*T*_min_ = 0.981, *T*_max_ = 0.990	*l* = −15→15
2593 measured reflections	3 standard reflections every 120 min
2281 independent reflections	intensity decay: 1%

### Refinement

**Table:**

Refinement on *F*^2^	Secondary atom site location: difference Fourier map
Least-squares matrix: full	Hydrogen site location: inferred from neighbouring sites
*R*[*F*^2^ > 2σ(*F*^2^)] = 0.067	H-atom parameters constrained
*wR*(*F*^2^) = 0.165	*w* = 1/[σ^2^(*F*_o_^2^) + (0.057*P*)^2^] where *P* = (*F*_o_^2^ + 2*F*_c_^2^)/3
*S* = 1.00	(Δ/σ)_max_ < 0.001
2281 reflections	Δρ_max_ = 0.19 e Å^−^^3^
157 parameters	Δρ_min_ = −0.14 e Å^−^^3^
Primary atom site location: structure-invariant direct methods	

### Special details

**Table:**

Geometry. All e.s.d.'s (except the e.s.d. in the dihedral angle between two l.s. planes) are estimated using the full covariance matrix. The cell e.s.d.'s are taken into account individually in the estimation of e.s.d.'s in distances, angles and torsion angles; correlations between e.s.d.'s in cell parameters are only used when they are defined by crystal symmetry. An approximate (isotropic) treatment of cell e.s.d.'s is used for estimating e.s.d.'s involving l.s. planes.
Refinement. Refinement of *F*^2^ against ALL reflections. The weighted *R*-factor *wR* and goodness of fit *S* are based on *F*^2^, conventional *R*-factors *R* are based on *F*, with *F* set to zero for negative *F*^2^. The threshold expression of *F*^2^ > σ(*F*^2^) is used only for calculating *R*-factors(gt) *etc*. and is not relevant to the choice of reflections for refinement. *R*-factors based on *F*^2^ are statistically about twice as large as those based on *F*, and *R*- factors based on ALL data will be even larger.

### Fractional atomic coordinates and isotropic or equivalent isotropic displacement parameters (Å^2^)

**Table:**

	*x*	*y*	*z*	*U*_iso_*/*U*_eq_	
O1	−0.3870 (7)	−0.0274 (2)	0.8647 (2)	0.0865 (10)	
O2	−0.2837 (8)	−0.1310 (3)	0.7632 (3)	0.1130 (12)	
O3	−0.0492 (8)	0.3592 (2)	0.7202 (2)	0.1056 (12)	
O4	0.2630 (7)	0.4310 (3)	0.5801 (2)	0.104	
N1	0.1072 (8)	0.3526 (3)	0.6413 (3)	0.0706 (10)	
N2	0.4396 (8)	0.3293 (3)	0.4457 (3)	0.0842 (11)	
H2A	0.4491	0.3936	0.4521	0.101*	
C1	−0.3968 (13)	−0.2145 (4)	1.0388 (4)	0.127 (2)	
H1A	−0.5267	−0.2746	1.0918	0.190*	
H1B	−0.3079	−0.1811	1.0788	0.190*	
H1C	−0.2379	−0.2511	1.0058	0.190*	
C2	−0.5738 (10)	−0.1193 (4)	0.9484 (4)	0.1006 (16)	
H2B	−0.7339	−0.0825	0.9819	0.121*	
H2C	−0.6696	−0.1538	0.9100	0.121*	
C3	−0.2502 (11)	−0.0448 (4)	0.7781 (4)	0.0809 (13)	
C4	−0.0655 (9)	0.0540 (3)	0.6944 (3)	0.0664 (11)	
C5	−0.0537 (8)	0.1582 (3)	0.7019 (3)	0.0592 (10)	
H5A	−0.1638	0.1678	0.7620	0.071*	
C6	0.1163 (8)	0.2486 (3)	0.6230 (3)	0.0584 (10)	
C7	0.2859 (8)	0.2413 (3)	0.5268 (3)	0.0588 (10)	
C8	0.2554 (10)	0.1315 (4)	0.5233 (3)	0.0820 (13)	
H8A	0.3503	0.1217	0.4612	0.098*	
C9	0.1030 (11)	0.0445 (4)	0.6018 (4)	0.0834 (14)	
H9A	0.1064	−0.0264	0.5961	0.100*	
C10	0.5976 (13)	0.3193 (4)	0.3427 (4)	0.129 (2)	
H10A	0.8040	0.2821	0.3610	0.155*	
H10B	0.4927	0.2659	0.3281	0.155*	
C11	0.6104 (14)	0.4292 (5)	0.2425 (4)	0.138 (2)	
H11A	0.7199	0.4821	0.2561	0.165*	
H11B	0.4046	0.4674	0.2246	0.165*	
C12	0.7633 (11)	0.4158 (4)	0.1409 (3)	0.1116 (17)	
H12A	0.7768	0.4944	0.0758	0.167*	
H12B	0.6465	0.3692	0.1229	0.167*	
H12C	0.9645	0.3752	0.1591	0.167*	

### Atomic displacement parameters (Å^2^)

**Table:**

	*U*^11^	*U*^22^	*U*^33^	*U*^12^	*U*^13^	*U*^23^
O1	0.082 (2)	0.0819 (19)	0.0924 (19)	−0.0227 (18)	−0.0007 (18)	−0.0351 (17)
O2	0.149 (3)	0.0806 (19)	0.122 (2)	−0.028 (2)	−0.029 (2)	−0.0475 (19)
O3	0.128 (3)	0.103 (2)	0.104 (2)	−0.035 (2)	0.038 (2)	−0.0672 (19)
O4	0.123	0.115	0.097	−0.056	0.021	−0.063
N1	0.068 (2)	0.076 (2)	0.0711 (19)	−0.031 (2)	0.0100 (19)	−0.0344 (17)
N2	0.073 (3)	0.088 (2)	0.074 (2)	0.010 (2)	0.006 (2)	−0.0304 (18)
C1	0.133 (5)	0.088 (3)	0.104 (3)	0.007 (4)	0.004 (4)	−0.007 (3)
C2	0.070 (4)	0.089 (3)	0.126 (4)	−0.012 (3)	−0.017 (3)	−0.033 (3)
C3	0.079 (4)	0.063 (3)	0.094 (3)	0.001 (3)	−0.041 (3)	−0.025 (3)
C4	0.058 (3)	0.066 (2)	0.084 (3)	0.016 (2)	−0.030 (2)	−0.041 (2)
C5	0.050 (3)	0.065 (2)	0.067 (2)	0.002 (2)	−0.010 (2)	−0.034 (2)
C6	0.053 (3)	0.071 (2)	0.059 (2)	0.005 (2)	−0.013 (2)	−0.037 (2)
C7	0.043 (2)	0.064 (2)	0.054 (2)	0.021 (2)	−0.0114 (19)	−0.0212 (19)
C8	0.092 (4)	0.090 (3)	0.068 (3)	0.036 (3)	−0.019 (3)	−0.049 (2)
C9	0.104 (4)	0.069 (3)	0.089 (3)	0.014 (3)	−0.023 (3)	−0.048 (2)
C10	0.132 (4)	0.115 (4)	0.088 (3)	0.026 (3)	0.038 (3)	−0.026 (3)
C11	0.155 (5)	0.133 (4)	0.091 (3)	0.031 (4)	0.014 (4)	−0.041 (3)
C12	0.106 (4)	0.132 (4)	0.066 (2)	0.012 (3)	0.005 (3)	−0.030 (3)

### Geometric parameters (Å, °)

**Table:**

O1—C3	1.326 (5)	C4—C9	1.400 (5)
O1—C2	1.446 (4)	C5—C6	1.373 (4)
O2—C3	1.223 (4)	C5—H5A	0.9300
O3—N1	1.211 (3)	C6—C7	1.432 (4)
O4—N1	1.188 (3)	C7—C8	1.432 (5)
N1—C6	1.436 (4)	C8—C9	1.304 (5)
N2—C7	1.326 (4)	C8—H8A	0.9300
N2—C10	1.505 (5)	C9—H9A	0.9300
N2—H2A	0.8600	C10—C11	1.393 (5)
C1—C2	1.443 (5)	C10—H10A	0.9700
C1—H1A	0.9600	C10—H10B	0.9700
C1—H1B	0.9600	C11—C12	1.500 (5)
C1—H1C	0.9600	C11—H11A	0.9700
C2—H2B	0.9700	C11—H11B	0.9700
C2—H2C	0.9700	C12—H12A	0.9600
C3—C4	1.488 (5)	C12—H12B	0.9600
C4—C5	1.372 (4)	C12—H12C	0.9600
			
C3—O1—C2	117.2 (3)	C5—C6—N1	115.8 (3)
O4—N1—O3	120.0 (3)	C7—C6—N1	121.9 (3)
O4—N1—C6	119.2 (3)	N2—C7—C6	123.9 (4)
O3—N1—C6	120.8 (3)	N2—C7—C8	123.4 (3)
C7—N2—C10	121.4 (4)	C6—C7—C8	112.6 (3)
C7—N2—H2A	119.3	C9—C8—C7	124.2 (4)
C10—N2—H2A	119.3	C9—C8—H8A	117.9
C2—C1—H1A	109.5	C7—C8—H8A	117.9
C2—C1—H1B	109.5	C8—C9—C4	122.3 (4)
H1A—C1—H1B	109.5	C8—C9—H9A	118.8
C2—C1—H1C	109.5	C4—C9—H9A	118.8
H1A—C1—H1C	109.5	C11—C10—N2	114.4 (4)
H1B—C1—H1C	109.5	C11—C10—H10A	108.7
C1—C2—O1	111.7 (4)	N2—C10—H10A	108.7
C1—C2—H2B	109.3	C11—C10—H10B	108.7
O1—C2—H2B	109.3	N2—C10—H10B	108.7
C1—C2—H2C	109.3	H10A—C10—H10B	107.6
O1—C2—H2C	109.3	C10—C11—C12	113.1 (4)
H2B—C2—H2C	107.9	C10—C11—H11A	109.0
O2—C3—O1	123.7 (4)	C12—C11—H11A	109.0
O2—C3—C4	121.8 (5)	C10—C11—H11B	109.0
O1—C3—C4	114.4 (4)	C12—C11—H11B	109.0
C5—C4—C9	116.8 (4)	H11A—C11—H11B	107.8
C5—C4—C3	122.8 (4)	C11—C12—H12A	109.5
C9—C4—C3	120.4 (4)	C11—C12—H12B	109.5
C4—C5—C6	121.8 (3)	H12A—C12—H12B	109.5
C4—C5—H5A	119.1	C11—C12—H12C	109.5
C6—C5—H5A	119.1	H12A—C12—H12C	109.5
C5—C6—C7	122.2 (3)	H12B—C12—H12C	109.5
			
C3—O1—C2—C1	87.3 (5)	C5—C6—C7—N2	176.3 (4)
C2—O1—C3—O2	3.0 (6)	N1—C6—C7—N2	−2.0 (6)
C2—O1—C3—C4	179.2 (3)	C5—C6—C7—C8	0.2 (5)
O2—C3—C4—C5	172.1 (4)	N1—C6—C7—C8	−178.1 (3)
O1—C3—C4—C5	−4.1 (6)	N2—C7—C8—C9	−179.4 (4)
O2—C3—C4—C9	−6.9 (7)	C6—C7—C8—C9	−3.2 (6)
O1—C3—C4—C9	176.9 (4)	C7—C8—C9—C4	4.6 (7)
C9—C4—C5—C6	−0.4 (6)	C5—C4—C9—C8	−2.6 (6)
C3—C4—C5—C6	−179.4 (4)	C3—C4—C9—C8	176.5 (4)
C4—C5—C6—C7	1.5 (6)	C7—N2—C10—C11	148.5 (5)
C4—C5—C6—N1	179.9 (3)	N2—C10—C11—C12	−178.8 (4)

### Hydrogen-bond geometry (Å, °)

**Table:**

*D*—H···*A*	*D*—H	H···*A*	*D*···*A*	*D*—H···*A*
N2—H2A···O4	0.86	2.02	2.635 (5)	128
N2—H2A···O4^i^	0.86	2.55	3.324 (6)	150

Symmetry codes: (i) −*x*+1, −*y*+1, −*z*+1.
